# The effects of weather on physical activity and sedentary behaviour in older adults

**DOI:** 10.3389/fspor.2024.1468911

**Published:** 2025-01-20

**Authors:** Kathryn M. Crosby, Brittany Adams, Elizabeth Zambrano Garza, Mathieu L. Bourbonnais, Melanie Fenton, Christiane Hoppmann, Jennifer M. Jakobi

**Affiliations:** ^1^ Aging in Place Research Cluster, University of British Columbia Okanagan, Kelowna, BC, Canada; ^2^ School of Health & Exercise Sciences, University of British Columbia Okanagan, Kelowna, BC, Canada; ^3^ Edwin S.H. Leong Centre for Healthy Aging, The University of British Columbia, Vancouver, BC, Canada; ^4^ Department of Earth, Environmental, and Geographic Sciences, University of British Columbia Okanagan, Kelowna, BC, Canada; ^5^ Department of Psychology, The University of British Columbia, Vancouver, BC, Canada

**Keywords:** exercise, environment, climate, temperature, aging, sedentary

## Abstract

**Background:**

Many older adults do not meet current physical activity (PA) guidelines, and this might be influenced by environmental factors (e.g., temperature). The purpose of this study was to determine how various weather variables influence light PA (LPA), moderate-vigorous PA (MVPA), and sedentary behaviour (SB), steps, and time spent walking outdoors.

**Methods:**

Fifty community-dwelling older adults completed the 10-day assessment period, using a Fitbit fitness tracker, Global Position System (GPS), and completing self-report questionnaires. Forty participants were included in the analysis. LPA, MVPA, and SB were calculated using heart rate and multilevel models were used to assess their association with weather variables.

**Results:**

Number of steps was positively associated with self-reported health status (0.18, *p* < 0.01). Increased age was associated with less time spent engaging in LPA (total and outdoors), reduced steps, increased time spent sedentary, and less time spent walking outdoors. LPA outdoors was positively associated with mean temperature. Freeze thaw cycles were associated with time spent walking outdoors. Females spent more time in total LPA and LPA indoors, and as age increased total LPA and LPA outdoors decreased.

**Conclusions:**

Older adults located in a dry (∼690 mm precipitation annually) region with warm winters and hot summers (−5°C–30°C) seemingly choose indoor activities. Females tend to participate in indoor LPA, and as age increases the time spent in outdoor LPA decreases.

**Implications:**

Consideration needs to be given to designing indoor PA interventions, with a focus on increasing MVPA for older adults.

## 1 Introduction

Across the globe people are living longer than they ever have before. Yet, health, functional independence, and quality of life have not kept pace with this trend (1). A substantial body of literature has illustrated the beneficial effects that physical activity (PA) has on health and well-being (2). Research demonstrates that regular participation in PA and reduction of sedentary behaviour (SB) is vital for healthy aging, as it lowers the risk of chronic disease and premature death (3, 4) and improves physical and cognitive function and overall quality of life (5). SB is defined as “any waking behavior characterized by an energy expenditure ≤1.5 metabolic equivalents (METs), while in a sitting, reclining or lying posture” (6). The World Health Organization (WHO) recommends older adults achieve 150 min of moderate PA or 75 min of vigorous aerobic PA per week (7), but previous studies have found that many older adults do not meet PA guidelines (8). A recent review reported that globally 18.8% of males and 24.5% of females aged 60–69 years do not meet PA guidelines, with percentages increasing in older age groups (8). PA participation tends to decrease as age increases (8). More specifically, older adults tend to participate in lower amounts of moderate PA and vigorous PA as they age (9). This might be the result of age-related physical changes including reduced muscle mass and strength, and impaired balance and gait, all of which affect mobility (10). Light PA (LPA) is an activity that is classified as less than 3 METs and moderate-vigorous PA (MVPA) is an activity that is classified as 3 or more METs (11). Examples of LPA include walking, housework, and light weight training, and examples of MVPA include running, biking, and swimming.

Older adults experience multiple barriers to participating in regular PA. Older Canadians self-reported in one study that the effects of aging (e.g., pain, arthritis), safety concerns from family, and fear of falling due to inclement weather (e.g., ice) were the most common barriers influencing their PA participation (12). Older adults may perceive a lack of opportunities to safely participate in PA during the cold of winter months and heat of summer. Weather variations, with extremes of cold and heat temperatures, coupled with precipitation and daylight hours present perceived dangers and obstacles that challenge safety and comfort for PA participation in older adults, and might contribute to adapting the location of engagement, to an indoor location, as many use walking as their main mode of PA (13). The built environment (walkability, access to destinations, public transport) also affects PA participation (14), and variation between days might play a role in facilitating or preventing PA during various weather events or extremes.

Previous research with older adults has found MVPA duration to be positively associated with temperature, and SB to be negatively associated with temperature (15, 16). Precipitation has been reported to be negatively associated with MVPA (17) and positively associated with SB in older adults (18). Daylight hours are positively associated with MVPA (18) and negatively associated with SB in older adults (15). A recent scoping review outlined a need for studies to investigate the effects of snow accumulation on PA and SB (19). This review also stated the need to include LPA in future studies. This is due to the benefits of LPA acting as an alternative to replace SB as older adults spend the majority of their PA time in the LPA zone (20). Currently, there is a knowledge gap on the relationship between LPA relative to ambient temperature, precipitation, and daylight hours, yet all these factors influence MVPA (19).

In northern climates, like those experienced throughout much of Canada, many older adults do not meet the national PA guidelines (21). There is a paucity of research that considers the variation of yearly weather conditions on PA participation within a region. To fully understand weather conditions the LPA and MVPA activity completed indoors and outdoors needs to be evaluated, and time spent walking outdoors segregated from household chores to consider walking LPA outdoors. A previous study looking at individual perceptions of weather and PA behaviours reported rain as the predominant weather variable to increase odds of indoor PA participation (22). Walking is the most common LPA mode in which older adults participate, therefore, understanding how various weather variables influence walking PA, which would be distinct from other forms of in-home LPA is necessary (13).

The effects of climate change may also contribute to rapid temperature changes which can impact both the outdoor and indoor environments. Two unique weather variables recently associated with climate change include more freeze-thaw cycles during the winter and more frequent tropical nights during the summer months. Freeze-thaw cycles occur when the temperature is above freezing (0°C), then drops below freezing, followed by a return to above 0°C which may cause icy surfaces that are perceived dangerous for walking. Tropical nights occur when the temperature does not drop below 20°C making PA difficult, both outside and indoors, if not climate-controlled. Investigating the influence of both winter and summer climate variables on device-based measured LPA and MVPA is important to understand how the weather might influence older adults achieving the recommended PA guidelines. This knowledge would inform where (indoor or outdoor) the activity is undertaken, assist in understanding approaches to built environments, and use of outdoor spaces to better define the location of interventions to support the type and intensity of PA to be most effective for older adults.

The purpose of this study was to examine the effects of various weather variables on minutes spent engaging in walking, LPA, MVPA, and SB time. This investigation also examined the influence of weather variables on minutes spent engaging in PA indoors and outdoors, and time spent walking outdoors. To explore the contributions of various weather factors that often occur independently of each other (e.g., freeze-thaw cycle and tropical nights do not simultaneously occur) on different PA measures (e.g., indoor and outdoor, walking and non-walking PA) multiple unique models were necessary. The overarching hypothesis tested across models is that inclement weather would negatively impact outdoor walking, and not outdoor LPA. We also expected a positive contribution of inclement weather to increased indoor PA.

## 2 Methods

### 2.1 Participants

Fifty-five participants were recruited from the Thompson-Okanagan region within British Columbia, Canada through local advertisements, University of British Columbia Embrace Aging events, and through REACH BC, a province-wide health research recruitment strategy. Inclusion within the study required participants to be aged 60 years and older, reside in the Thompson-Okanagan area, be able to read newspaper-sized print, have access to a regular internet connection, able to leave their homes and walk in their neighborhood independently without assistance. All participants provided written consent and the study was approved by the University of British Columbia Ethics Board (study number: H21-01624). This study was performed in accordance with the Declaration of Helsinki.

### 2.2 Study design and procedures

Data collection occurred between October 2021 and October 2022, and each participant was measured for one data collection period that comprised 10 consistent days of monitoring. Participants wore a fitness tracker (Fitbit Charge 4, San Francisco California, USA) on their non-dominant wrist to record heart rate and daily step count. The Global Positioning System (GPS) watch (Garmin Forerunner 55, Olathe Kansas, USA) was worn on their dominant wrist to record GPS data of movement throughout the day, for each day of the 10-day assessment period. Hand dominance was determined by participant self-report. An Apple iPad Mini 5th or 6th generation (Apple Inc., Cupertino California, USA) was used by the participants to complete the baseline questionnaire using the “Qualtrics Surveys” app (Version 17.1.0, Qualtrics Labs, Inc., Provo, Utah). Participants independently completed the baseline questionnaire before the 10-day assessment which collected demographic information (e.g., age, sex). Responses to the questionnaires were stored on the iPad and uploaded after completion of data collection to a password-secure institutional computer system.

Participants were screened and informed about the study through a telephone call. All meetings occurred in public and in outdoor spaces to align with COVID-19 protocols. A designated research team member met with each participant, in-person, twice; first at the baseline session, and second after the 10-day data collection period was completed. Participants received a telephone call at the mid-point (day 5 or 6) of the data-collection period to ensure that all equipment was working properly and that the participant was following the prescribed protocol. In addition, participants were encouraged to call the researcher throughout the study if they had questions or required assistance with the recording equipment.

At the baseline meeting participants with the researcher re-reviewed the previously sent study procedures and provided written consent. Upon consent, baseline assessment occurred over 30 min–3 h, depending on the participant. Information on sociodemographic characteristics (sex, age) and health status was collected in the baseline survey. Health status was determined by participants self-reporting their overall health on a scale from 1 to 5, where 1 represents poor health and 5 represents excellent health.

During the baseline meeting, participants were given a “daily schedule calendar” that instructed them on what to do each morning, throughout their day, and each evening for each of the 10 days. During the baseline meeting, participants were given the opportunity to practice with each device (Garmin and Fitbit watches, iPad) while the researcher was present. They were also given a “participant manual” that included instructions (written and photographed) on how to set up, activate, download, and recharge the monitoring equipment each day. Participants were provided chargers and directed to charge all three devices each night while they slept, and instructed to begin wearing the devices, upon waking, throughout the day, and until they went to bed each night. All participants received a $50 CDN grocery gift card for their participation in the study.

The participants were asked to sync the Fitbit device with the iPad each evening. To collect the timestamped coordinate data, they were instructed to start a “Walk” activity on the Garmin device each morning and end it each night. A “Walk” activity ran from the beginning to the end of a participant's day and allowed the Garmin device to collect GPS data of the participant's movements throughout the day. The Garmin device was set to record GPS data points on “Smart Recording” mode, meaning data points are taken when there are changes in direction, speed, heart rate, or elevation. The frequency of GPS fixes was generally recorded by the device every 1 to several seconds. The number of GPS points varied for each participant, each day (1,157–45,295). It depended on several factors including time spent asleep, loss of signal, and correct use of the device (i.e., a participant forgetting to start a “Walk” activity). Garmin data was stored on the device for the study period and downloaded by researchers after devices were retrieved from participants.

#### 2.2.1 Data processing

The original TCX files from the Garmin GPS devices were downloaded individually for each participant, and any records that lacked either a coordinate fix or a measure of heart rate were removed. Then, for each record, the distance (in meters) and time (in seconds) between that fix and the subsequent fix were calculated. Outliers were addressed by removing records containing speeds of >50 m/s and distances of >30 m between consecutive fixes (23). Records containing duplicate timestamps were also removed. For example, if two GPS fixes with identical timestamps occurred (i.e., multiple fixes were recorded at the same point in time), the first fix to be recorded was kept and any other fixes with the timestamp were removed. Periods in which no fixes were recorded for at least 60 min were classified as non-wear time (24, 25).

After the time series data had been prepared and outliers were removed, each record was designated as “LPA”, “MVPA”, or “SB” according to the heart rate thresholds identified for each participant. Each record was then designated as occurring either indoors or outdoors by overlaying the time series data with building footprints obtained from a database (https://github.com/microsoft/CanadianBuildingFootprints) or the City of Kelowna for GPS data collected within Kelowna (26) Records were only considered to be outdoors if at least five consecutive fixes occurred outside of a building footprint; this was to prevent GPS positional errors from artificially inflating the true amount of time participants spent outdoors. The GPS time series data were then grouped and summarized by both participant and date to calculate the total number of minutes spent engaging in LPA, MVPA, SD, and time spent walking outdoors each day for each participant. Following statistical analysis, daily minutes were converted to hours for readability.

#### 2.2.2 Physical activity and sedentary behaviour

The time spent engaging in LPA, MVPA, and SB was calculated using the heart rate values associated with the time series data of each participant. Heart rate values throughout the day were collected on the Garmin device so that they could be easily time-matched with the GPS data to match location and calculate intensity. The resting heart rate (RHR) was collected using the Fitbit device by collecting all daily resting heart rate values recorded across the study period and calculating their mean value. This was done due to missing data at the onset of the recording day to quantify and calculate from the GPS device RHR (e.g., time to associate device to satellites was insufficient during the sitting period at the beginning of the day). Heart rate reserve (HRR) was then obtained by subtracting the participant's resting heart rate from their maximum heart rate, and threshold values determined from evidence-informed physical activity guidelines (27). Maximum heart rate was calculated by subtracting their age at the time of the study from 220. HRR thresholds are used to calculate the total daily time in minutes spent in SB (HR <20% of HRR), in LPA (HR is 20%–39% of HRR), and in MVPA (HRR is ≥40%).

#### 2.2.3 Time spent walking outdoors

For each participant's GPS time series, days containing < nine hours of data and participants with < four days of data were excluded from the analysis. For the remaining data, records containing missing coordinate data were removed, and outliers were addressed by removing records containing speeds of >50 m/s and distances of >30 m between consecutive fixes, as described above (23). The GPS timeseries data for each individual and day were then segmented using the “get-clusters” tool in the “places” package (28) in R Core Team (2022). This approach clusters GPS coordinates into “stops”, or places of interest, and classifies all non-stop fixes as being in-transit. Clusters of records representing places of interest were identified where consecutive fixes with speeds of <0.5 m/s occurred for at least 2 min within a radius of 150 m. GPS segments not considered a part of a cluster were then screened to identify trips that occurred outside of the participant's home. A segment was considered a trip if it lasted ≥3 min, was ≥100 m in length and exhibited a mean speed of ≥1 km/h (29). All other segments were classified as not being part of a trip. Walking trips were identified if they lasted ≥3 min long and was ≥100 m in length and exhibited a mean speed of ≥1 km/h (29). All other segments were classified as not being part of a trip.

The transport mode of each trip was classified using the 90th percentile speed of each segment (29). Trips with a 90th percentile speed of ≥1 km/h and <10 km/h were classified as walking, trips with a 90th percentile speed of ≥10 km/h and <25 km/h were classified as biking, and trips with a 90th percentile speed of ≥25 km/h were lastly classified as vehicular. The length of time spent walking outdoors each day was then calculated by overlaying the GPS time series data with building footprints.

#### 2.2.4 Steps

Number of steps per day was measured using the Fitbit Charge 4 device. Fitbit devices worn on the wrist have been validated against research-based trackers for measuring daily steps in community-dwelling older adults (30) and to assist in verifying ambulatory behaviour step counts during given periods of interest were compared with GPS, and heart rate data.

#### 2.2.5 Weather variables

The weather variables investigated included mean temperature over 24 h, daily totals (i.e., sum) of daylight hours (total hours between sunrise and sunset), precipitation, and snow accumulation, and evidence of freeze-thaw events and tropical nights. Climate data were downloaded from Environment and Climate Change Canada (31) by selecting the active weather station nearest to a participant's residence that recorded daily minimum and maximum temperature, total precipitation, and ground snow accumulation. Days in which the minimum temperature did not fall below 20°C were defined as a tropical night. A freeze-thaw event occurs when the minimum temperature falls to 0°C or below and the maximum temperature exceeds 0°C on the same day.

To match daily weather data with the summarized time series data, the date ranges in which time series data was collected for each participant were first identified. Daily weather data were then downloaded from Environment and Climate Change Canada and merged with the summarized time series data by date for each participant according to the weather station geographically nearest to the participants' residence. Lastly, for each participant, the daily number of steps, time spent sedentary, and resting heart rate were extracted from the original FitBit csv files and merged with the summarized time series data according to date.

### 2.3 Statistical analyses

Multilevel models (R lme4 package v.1.1-30) (32) using restricted maximum likelihood estimation (32) were used to accommodate the 2-level hierarchical structure of the data (i.e., days nested within individuals) using R (v.4.1.1). For each of LPA and MVPA, three models were fit to examine weather (see above section on weather variables), on daily time spent (minutes) in total LPA (Model 1), indoors (Model 2) and outdoors (Model 3), as well as total MVPA (Model 4), indoors (Model 5) and outdoors (Model 6) controlling for age, sex, health, day of the week (i.e., weekend vs. weekday), daylight hours. Separate models for SB (Model 7), total steps per day (Model 8), and the total time spent walking outdoors (Model 9) were also considered.

Intraclass Correlation Coefficients (ICC) were calculated using a random-intercept model to assess the proportion of variance in the outcome variable attributed to between-person variability. ICCs were 0.41 for Model 1 (total LPA), 0.50 for Model 2 (LPA Indoors), 0.33 for Model 3 (LPA Outdoors), 0.43 for Model 4 (total MVPA), 0.39 for Model 5 (MVPA Indoors), 0.36 for Model 6 (MVPA Outdoors), 0.45 for Model 7 (SB), 0.46 for Model 8 (steps), and 0.26 for Model 9 (time spent walking outdoors). This indicates that PA varied between individuals as well as on a day-to-day basis within the same individual. A simulation-based power analysis (33) indicated our sample size allowed us to detect small main effects at Level 1 [minimum detectable effect size (MDES) = 0.20; see Table 5 in (33) and Level 2 (MDES) = (MDES) = 0.57; see Table 6 in (33)] with 80% power. All day-level variables were separated into daily effects (person-centered) and overall effects (grand-mean-centered person means). Daily variables capture intraindividual variation (e.g., within-person effects) whereas person-level (overall) variables examine interindividual variation (e.g., between-person effects). Sex, freeze thaw, tropical nights, and time of week were dichotomous variables in all models. Additionally, age and health were grand-mean centered. Participant characteristics were reported as mean ± SD.

## 3 Results

**Figure 1:**
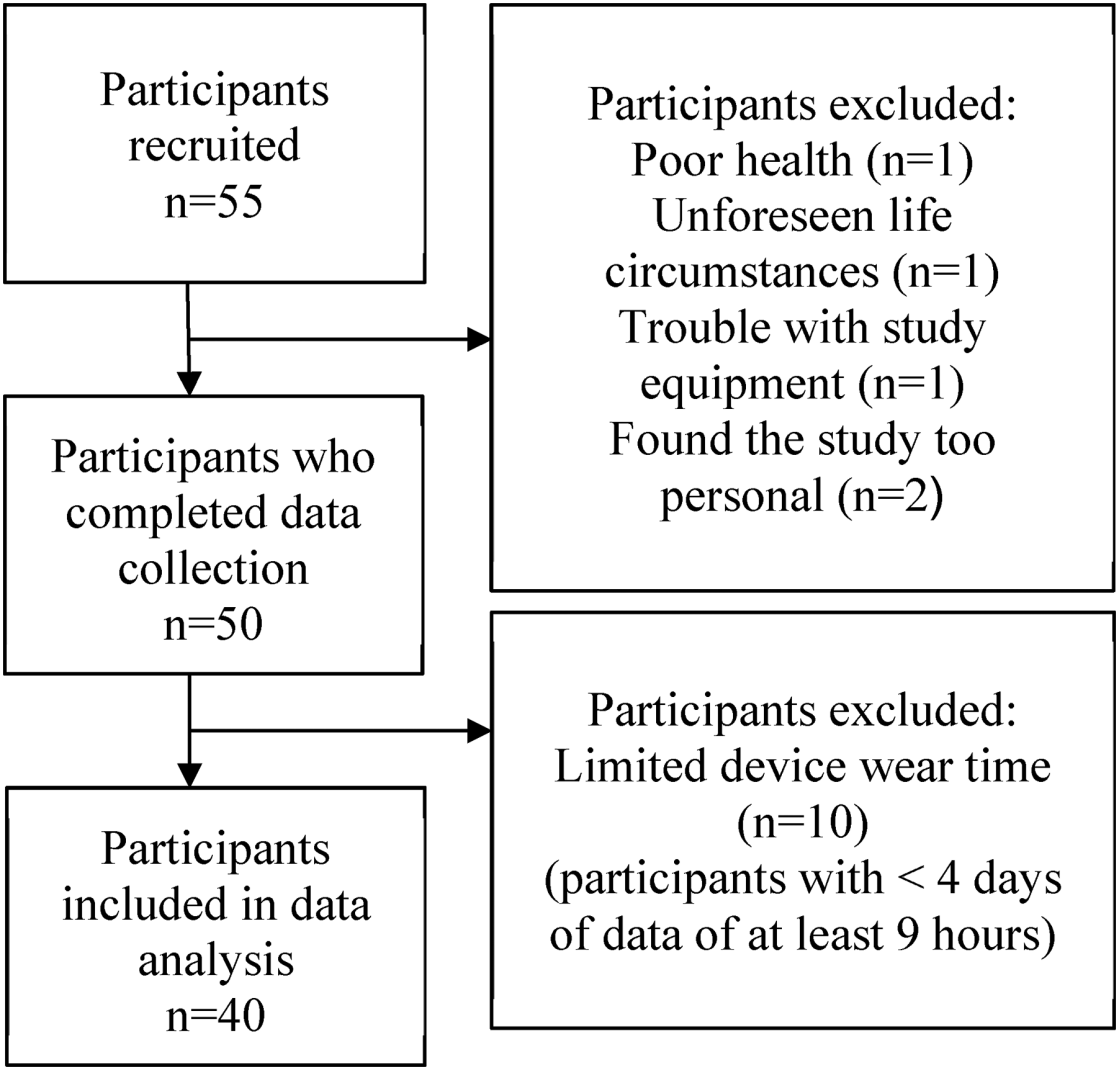
Flow diagram of participant recruitment and inclusion.

Forty participants were included in the analysis (69.3 ± 6.25 years, Table 1), and the average wear time of the devices was 12.73 ± 1.62 h. Figure 1 depicts participant recruitment and inclusion in the study.

**Table 1:** Age-Sex characteristics of participants (*N* = 40).

Age category	Female	Male
60–69 years	12	7
70–79 years	11	8
80+ years	2	0

The weather conditions across the study period are identified in Table 2, and align with those expected for the Interior of British Columbia (31). Mean participant self-reported health status was 3.87 ± 0.80 (range 1–5). Health status was negatively correlated with age and sex (both: −0.26, *p* = <0.01), meaning that older participants and males were more likely to rate their overall health lower. The mean time spent engaging in LPA was 196.82 ± 98.38 min per day (indoor LPA 116.36 ± 78.05 min per day; outdoor LPA 80.46 ± 57.57 min per day). The mean time spent engaging in MVPA was 74.71 ± 70.05 min per day (indoor MVPA 30.00 ± 38.21 min per day; outdoor MVPA 44.71 ± 48.53 min per day). Out of 40 participants, 18 participants (45%) exceeded the recommended 150 min of MVPA per week. The mean time spent sedentary was 492.50 ± 156.21 min per day. Mean number of steps was 9,861.85 ± 5,539.80 per day. The mean time spent walking outdoors was 25.26 ± 35.99 min per day. Number of steps was positively associated with health status (0.18, *p* < 0.01). Minutes per day were converted to hours, and presented in Table 3.

**Table 2:** Summary statistics for daily weather variables across all participants (*N* = 40).

Variable	Mean	SD	Min	Max	Range
Daylight hours	11.96	2.84	8.10	16.35	8.10–16.35
Maximum temperature (°C)	16.08	12.29	−6.5	38.7	−6.5–38.7
Mean temperature (°C)	10.92	10.54	−11.3	29.3	−11.3–29.3
Minimum temperature (°C)	5.75	9.09	−16.1	22.0	−16.1–22.0
Total precipitation (mm)	0.96	2.45	0	17.4	0–17.4
Snow accumulation (cm)	1.64	4.34	0	22	0–22

Note: “Daylight hours” refers to the total number of hours between dawn and dusk. “Snow accumulation” refers to the recorded height of snow on the ground for a given day. Climate data obtained from Environment and Climate Change Canada (31).

**Table 3:** Summary data in hour for physical activity measures (*N* = 40).

Variable	Mean	SD
LPA total time	3.26	1.63
LPA indoor time	1.93	1.30
LPA outdoor time	1.33	0.95
MVPA total time	1.23	1.17
MVPA indoor time	0.50	0.63
MVPA outdoor time	0.73	0.80
Sedentary time	8.20	2.60
Time spent walking outdoors	0.42	0.60
Steps	9,861.85	5,539.80

Note: Time reported in hours. Data in text and for statistical analysis conducted in minutes.

### 3.1 LPA

Table 4 shows three models of LPA, including total LPA (Model 1), LPA indoors (Model 2), and LPA outdoors (Model 3). The mean temperature was significantly associated with LPA outside only at the day level, suggesting that individuals engaged in more LPA outside on days when the daily average temperature was higher (*b* = 2.33, *p* = .007), overall levels did not reach statistical significance (*b* = −1.55, *p* = .193). There was a significant effect of LPA indoors with the study day (*b* = −2.31, *p* = 0.027). Indicating that as the study day progresses (number of days of participation) the amount of LPA indoors decreases. Precipitation, snow, daylight, freeze-thaw cycle, and tropical nights did not emerge as significant predictors of LPA (non-significance ranged from 0.073 to 0.099).

**Table 4:** Results for total LPA (model 1), LPA indoors (model 2), and LPA outdoors (model 3).

Predictors	Model 1			Model 2			Model 3		
	Estimates	CI	*p*	Estimates	CI	*P*	Estimates	CI	*P*
(Intercept)	162.44	124.94–199.95	**<0** **.** **001**	87.98	56.82–119.13	**<0** **.** **001**	74.49	52.27–96.70	**<0** **.** **001**
Mean temp daily	2.20	−0.36–4.76	0.092	−0.12	−2.00–1.75	0.897	2.33	0.63–4.03	**0** **.** **007**
Mean temp overall	3.28	−0.80–7.36	0.115	1.75	−1.73–5.23	0.324	1.55	−0.79–3.89	0.193
Precipitation daily	0.89	−2.50–4.28	0.608	2.28	−0.21–4.76	0.073	−1.39	−3.64–0.87	0.227
Precipitation overall	2.88	−13.82–19.58	0.735	−0.80	−14.95–13.34	0.911	3.68	−5.98–13.33	0.454
Snow daily	−2.07	−7.35–3.20	0.440	−0.71	−4.58–3.16	0.718	−1.36	−4.86–2.15	0.448
Snow overall	−0.73	−6.56–5.09	0.805	0.13	−4.84–5.11	0.958	−0.85	−4.18–2.48	0.616
Daylight daily	−27.10	−82.77–28.57	0.339	−20.14	−60.99–20.70	0.333	−6.99	−44.02–30.05	0.711
Daylight overall	−9.77	−23.52–3.98	0.163	−8.54	−20.29–3.20	0.153	−1.27	−9.13–6.59	0.750
Freeze thaw	5.17	−18.72–29.07	0.671	6.41	−11.18–24.00	0.474	−0.86	−16.68–14.97	0.915
Tropical night	−12.26	−61.63–37.10	0.625	0.45	−35.99–36.88	0.981	−12.93	−45.51–19.65	0.435
Study day	−1.89	−4.67–0.89	0.183	−2.31	−4.35 to −0.27	**0** **.** **027**	0.41	−1.44–2.26	0.661
Age	−4.47	−8.10 to −0.84	**0** **.** **016**	−1.88	−4.98–1.22	0.233	−2.59	−4.67 to −0.52	**0** **.** **015**
Sex	71.62	28.08–115.16	**0** **.** **001**	62.86	25.66–100.06	**0** **.** **001**	8.56	−16.33–33.45	0.499
Health	−13.49	−41.44–14.46	0.343	−12.73	−36.61–11.16	0.295	−0.88	−16.86–15.09	0.913
Weekend	−0.91	−17.19–15.37	0.913	1.46	−10.49–13.41	0.810	−2.22	−13.04–8.61	0.687
Random effects									
σ^2^	4,896.45			2,634.45			2,167.65		
*τ*_00__ID_	3,435.24			2,612.77			1,059.45		
N _ID_	40			40			40		
Observations	363			363			363		
Marginal R^2^/Conditional R^2^	0.218/0.540			0.231/0.614			0.105/0.399		

Note: Bolded *p*-values are significant at *p* < 0.05. Model entry of Sex (1 = female), freeze thaw (1 = occurred), tropical night (1 = occurred), weekend (1 = weekend day; Saturday or Sunday). Daily variables capture intraindividual variation (e.g., within-person effects) whereas person-level (overall) variables examine interindividual variation (e.g., between-person effects).

### 3.2 Sex

Sex was found to be associated with total LPA (*b* = 71.62, *p* = 0.001, Model 1), LPA indoors (*b* = 62.86, *p* = 0.001, Model 2), and time spent sedentary (*b* = −88.72, *p* = 0.022, Model 7). Females spent more time engaging in total LPA and LPA indoors. Males spent more time sedentary compared to females.

### 3.3 Age

Age was found to be associated with total LPA (*b* = −4.47, *p* = 0.016, Model 1), LPA outdoors (*b* = −2.59, *p* = 0.015, Model 3), SB (*b* = 6.43, *p* = 0.046, Model 7), steps (*b* = −320.64, *p* = 0.005, Model 8), and time spent walking outdoors (*b* = −1.77, *p* = 0.003, Model 9). Higher age was associated with lower total LPA, LPA outdoors, steps, and time spent walking outdoors. SB was positively associated with age.

### 3.4 MVPA

For the three models of MVPA, including total (Model 4), indoors (Model 5), and outdoors (Model 6) (Table 5), no weather variables were a factor in the total time spent engaging in total MVPA, MVPA indoors, and MVPA outdoors.

**Table 5:** Results for total MVPA (model 4), MVPA indoors (model 5), and MVPA outdoors (model 6).

Predictors	Model 4			Model 5			Model 6		
	Estimates	CI	*p*	Estimates	CI	*P*	Estimates	CI	*P*
(Intercept)	72.47	43.27–101.67	**<0** **.** **001**	27.70	12.53–42.87	**<0** **.** **001**	44.77	25.07–64.46	**<0** **.** **001**
Mean temp daily	0.50	−1.45–2.45	0.614	−0.31	−1.38–0.75	0.565	0.82	−0.62–2.26	0.263
Mean temp overall	−1.90	−5.10–1.30	0.243	−0.63	−2.27–1.01	0.452	−1.27	−3.38–0.84	0.236
Precipitation daily	0.13	−2.45–2.71	0.921	0.17	−1.23–1.58	0.808	−0.05	−1.95–1.86	0.959
Precipitation overall	−5.76	−18.81–7.29	0.386	−3.78	−10.49–2.94	0.270	−1.99	−10.64–6.67	0.652
Snow daily	−1.84	−5.85–2.17	0.367	−1.14	−3.33–1.05	0.307	−0.70	−3.67–2.26	0.641
Snow overall	0.31	−4.25–4.88	0.893	0.38	−1.96–2.72	0.749	−0.07	−3.07–2.93	0.962
Daylight daily	1.51	−40.84–43.87	0.944	12.59	−10.56–35.73	0.285	−11.18	−42.45–20.09	0.482
Daylight overall	3.54	−7.23–14.30	0.518	−1.35	−6.87–4.17	0.631	4.89	−2.19–11.97	0.175
Freeze thaw	0.18	−18.01–18.37	0.985	2.66	−7.27–12.58	0.599	−2.52	−15.91–10.86	0.711
Tropical night	21.91	−15.70–59.51	0.253	4.43	−16.06–24.91	0.671	16.66	−10.95–44.27	0.236
Study day	−1.36	−3.48–0.75	0.205	−0.51	−1.66–0.65	0.390	−0.86	−2.42–0.71	0.282
Age	−1.43	−4.27–1.41	0.323	−0.55	−2.00–0.91	0.463	−0.88	−2.75–0.99	0.357
Sex	9.83	−24.26–43.93	0.571	7.89	−9.59–25.37	0.375	2.00	−20.43–24.42	0.861
Health	7.32	−14.57–29.20	0.511	2.35	−8.88–13.57	0.681	4.98	−9.41–19.38	0.496
Weekend	6.52	−5.86–18.91	0.301	−2.08	−8.85–4.68	0.545	8.64	−0.50–17.79	0.064
Random effects									
σ^2^	2,833.36			846.18			1,545.14		
τ_00__ID_	2,125.64			547.42			884.17		
N _ID_	40			40			40		
Observations	363			363			363		
Marginal R^2^/Conditional R^2^	0.086/0.478			0.129/0.471			0.058/0.401		

Note: Bolded *p*-values are significant at *p* < 0.05. Model entry of Sex (1 = female), freeze thaw (1 = occurred), tropical night (1 = occurred), weekend (1 = weekend day; Saturday or Sunday). Daily variables capture intraindividual variation (e.g., within-person effects) whereas person-level (overall) variables examine interindividual variation (e.g., between-person effects).

### 3.5 Sedentary behaviour

No weather variables were a factor in time spent sedentary (non-significance ranged from 0.074 to 0.993) (Table 6; Model 7).

**Table 6:** Results for SB (model 7), steps (model 8), and time spent walking outdoors (model 9).

Predictors	Model 7			Model 8			Model 9		
	Estimates	CI	*p*	Estimates	CI	*P*	Estimates	CI	*P*
(Intercept)	537.86	473.37–602.34	**<0** **.** **001**	10,374.40	8,122.62–12,626.18	**<0** **.** **001**	28.15	15.22–41.07	**<0** **.** **001**
Mean temp daily	−2.87	−7.05–1.31	0.178	83.22	−59.94–226.39	0.254	0.68	−0.41–1.77	0.219
Mean temp overall	−3.74	−10.84–3.37	0.302	−0.04	−249.07–249.00	1.000	−0.31	−1.63–1.00	0.640
Precipitation daily	1.01	−4.53–6.55	0.721	−51.78	−240.73–137.17	0.590	0.61	−0.83–2.05	0.403
Precipitation overall	6.42	−22.54–35.38	0.663	429.35	−584.27–1,442.96	0.405	0.61	−4.87–6.08	0.827
Snow daily	7.85	−0.77–16.46	0.074	−244.65	−538.46–49.17	0.102	−2.20	−4.44–0.04	0.054
Snow overall	−0.41	−10.56–9.73	0.937	11.41	−344.22–367.04	0.950	1.04	−0.83–2.90	0.275
Daylight daily	51.78	−39.16–142.71	0.264	−2,565.77	−5,673.27–541.72	0.105	−17.76	−41.39–5.87	0.140
Daylight overall	11.36	−12.57–35.30	0.351	97.42	−741.86–936.70	0.820	0.41	−4.00–4.81	0.856
Freeze thaw	−0.56	−39.65–38.53	0.978	−403.20	−1,744.27–937.87	0.555	−11.57	−21.61 to −1.52	**0** **.** **024**
Tropical night	15.76	−65.09–96.61	0.702	1,099.30	−1,661.92–3,860.53	0.434	4.44	−16.17–25.04	0.672
Study day	1.68	−2.86–6.22	0.468	−72.54	−227.73–82.66	0.359	−0.38	−1.56–0.80	0.528
Age	6.43	0.11–12.75	**0** **.** **046**	−320.64	−541.99 to −99.28	**0** **.** **005**	−1.77	−2.94 to −0.61	**0** **.** **003**
Sex	−88.72	−164.53 to −12.91	**0** **.** **022**	−325.44	−2,984.61–2,333.73	0.810	−0.87	−14.82–13.09	0.903
Health	0.22	−48.45–48.89	0.993	690.05	−1,015.90–2,396.00	0.427	−2.06	−11.02–6.89	0.650
Weekend	0.60	−25.99–27.20	0.964	169.63	−738.04–1,077.30	0.713	5.31	−1.59–12.21	0.131
Random effects									
σ^2^	13,061.34			15,188,315.65			882.87		
τ_00__ID_	10,614.95			13,135,051.57			310.38		
N _ID_	40			40			40		
Observations	363			362			363		
Marginal R^2^/Conditional R^2^	0.129/0.519			0.152/0.545			0.133/0.359		

Note: Bolded *p*-values are significant at *p* < 0.05. Model entry of Sex (1 = female), freeze-thaw (1 = occurred), tropical night (1 = occurred), weekend (1 = weekend day; Saturday or Sunday). Daily variables capture intraindividual variation (e.g., within-person effects) whereas person-level (overall) variables examine interindividual variation (e.g., between-person effects).

### 3.6 Steps

There were also no weather variables as significant factors in number of steps (non-significance ranged from 0.102 to 0.958) (Table 6; Model 8).

### 3.7 Time spent walking outdoors

Table 6 shows the model of time spent walking outdoors (Model 9). Freeze-thaw cycles were found to be negatively associated with time spent walking outdoors (*b* = −11.57, *p* = 0.024). No other weather variables were a factor in time spent walking outdoors (non-significance ranged from 0.054 to 0.966).

## 4 Discussion

The purpose of this study was to understand the effects of various weather variables on minutes spent engaging in indoor and outdoor LPA, and MVPA, time spent sedentary, steps, and time spent walking outdoors. We assessed 40 community-dwelling older adults using GPS watches and fitness trackers to understand how weather impacts PA intensity, location and SB. There was a positive association between LPA outdoors and mean temperature at the day level indicating that older adults engaged in more outdoor LPA on days when the temperature was up relative to their individual average LPA. There were also associations of sex and age with multiple outcome measures.

The finding of a positive association between mean temperature and LPA outside aligns with previous literature that showed a similar association with total LPA duration (15). In the present paper, indoor and outdoor LPA were delineated. Herein, the association with mean temperature was only apparent with outdoor LPA. This indicates that older adults spend more time engaging in LPA outdoors as the temperature increases at the day level. Understanding the influence of temperature might assist community-based planning for older adults participation in LPA. Unlike a prior study from Iceland (15), that observed the influence of daylight time this effect was not observed in this study. This may be due to the highly active population in our study or the result of cultural and regional differences between Iceland and Western Canada (e.g., availability of all-season PA infrastructure, and weather conditions outside of daylight hours). Results from this and prior studies draw attention to the important consideration of the environment as a strong mediating factor in LPA of older adults. Albeit, the environmental factors will vary based upon the region, it remains evident that LPA and outdoor temperature are important components of PA to gain health benefits especially as many older adults struggle to engage in regular MVPA for various reasons [e.g., lack of motivation, perceived poor health, physical disability (34)].

Increased age was associated with less time spent engaging in LPA (total and outdoors), reduced steps, increased time spent sedentary, and less time spent walking outdoors. Relative to the general older adult population in Canada this study sample was slightly younger (69.3 ± 6.25 years) than the population reported in the Canadian Longitudinal Study on Aging (73.36 ± 5.82), which included over twenty thousand Canadian older adults (35). Self-reported health status was similar between the present study and the Canadian Longitudinal Study on Aging, with both samples reporting self-rated health as good to very good (35). The findings from this study align with established trends in the literature that PA participation decreases progressively with age (21). Overall, the majority of participants' LPA happened indoors and this was especially evident in the females. Females were found to engage in more LPA (indoors and total) and spend less time sedentary compared to males. A previous study reported similar findings (36). In contrast, a systematic review found that older male PA levels were generally higher than older females, although the difference was less pronounced in studies that used accelerometer-measured PA (21). Although the measurement tool and potentially gait speed (37) might have some influence on the detection of LPA our data suggest females seem to engage in greater amounts than males. The increased indoor LPA might indicate a preference for planned walking. Location- and activity-specific accumulation of LPA should be further explored as an important factor in older adults transitioning between independent and assistive living as a systematic review of PA in older adults reported that females are less likely to engage in leisure time PA (21). Acquiring LPA through household activities might be an important means to maintaining health in older females (38, 39), and this should be specifically considered in research relative to intentional walking.

A benefit of using Fitbit devices in this study is the introduction of PA trackers to older adults who might have been unfamiliar with the technology. Contrary to this is the limitation that Fitbit devices underestimate heart rate (approximately 3 bpm) (40), limiting robust determination of intensity. However, activity trackers, such as Fitbits, are accessible and widely used by individuals of all ages. Having access to PA data (e.g., steps) might encourage older adults to make positive changes to their PA behaviour, benefitting their overall well-being (41).

Time spent walking outdoors was negatively associated with freeze-thaw cycles, meaning that participants spent more time walking outdoors when a freeze-thaw cycle did not occur. A freeze-thaw cycle could mean that ice and/or slush is present which might act as a physical or perceived physical barrier to walking outdoors. Many older adults experience a fear of falling due to inclement weather (e.g., ice) which could reduce the likelihood of walking outdoors during a freeze-thaw cycle (12). Overall, outdoor walking accounted for a small portion of participants' PA, and engagement was even lower during periods of freeze-thaw cycles. Time spent walking may have been underestimated if many brief walking trips occurred in the home or in instances where the GPS position might have been miscalculated arising from signal loss. What remains clear is extremes of cold and heat are often identified as barriers, and although these remain necessary comfort considerations, the “shoulder seasons” which have fluctuating temperatures are also strong factors. Weather variables need to be dissociated from seasons and given further consideration when designing formalized PA programs.

There were no significant effects of weather variables on MVPA (total, indoors, outdoors). This aligns with a recent scoping review that found no associations between MVPA duration and temperature, precipitation, and snow cover (19). However, other studies have found positive associations with temperature (15) and negative associations with precipitation (17, 42). One reason we, and others, might have not found any relationship between weather variables and MVPA could be self-selection bias, or the sample size. Meaning that individuals choosing to participate in this study might tend to be more active and less deterred by poor weather compared to the general population. It might also be due to the region of data collection. Precipitation may not impact PA significantly in a semi-arid climate. The region is also well-known for winter activities (e.g., downhill and cross-country skiing, snowshoeing) and the accessibility to outdoor activities in a temperate winter climate might contribute to maintaining modest levels of PA. Future work will incorporate qualitative data (daily logs and photovoice data) on daily activities collected during this study to better understand the motivating factors for engaging in PA at different times of the year.

A strength and novelty of this study was the pool of highly active participants. Many of the participants are meeting if not exceeding the PA guidelines (43). It seems that PA and SB might not be impacted by changes in weather variables in highly active older adults. Future studies need to objectively quantify the fitness and health status of participants. Those committed to engaging in regular exercise may be less influenced by weather than those who are less active and engage in recreational PA. This potential dichotomy of older adult participation in PA requires study. Another strength of this study is the use of GPS data and identifying walking relative to general LPA, as well as the location (indoor and outdoor) of walking PA. This novel approach enables an understanding of how weather influences the location of PA.

### 4.1 Limitations and future directions

There were no associations between PA, SB, steps, and time spent walking outdoors with precipitation, snow, and daylight hours and only one outcome measure (LPA outdoors) was associated with mean temperature. GPS position error must be considered, as time spent engaging in PA indoors vs. outdoors might not be exact as the GPS hops might lead to inaccuracies in the specific identification of indoor relative to outdoor e.g., facility with indoor and outdoor walking areas. A strength of this study is acquiring building footprints to overlay and verify location to minimize this error, yet it remains a potential consideration in GPS acquisition, and data interpretation. We found no overall effects of temperature on PA, which may be due to fluctuations within the 10-day collection period. Additionally, most of these relations are time-based variables and a time-of-day effect could have been present. Future studies should compare PA and SB to hourly weather data to assess the effects of weather variables directly with activity and intensity. This could be accomplished by programming PA monitoring devices to begin tracking at a specific start time so that PA can be easily time-locked to hourly weather data from local stations.

Participants encountered equipment challenges. A limitation of 10 days of in-home self-setup was the multiple steps to begin daily data collection. Many older adults were not familiar with the devices which resulted in a loss of data. Attempts were made to reduce this data loss by providing participants with written and pictured instructions for the devices and having a research assistant available in the mornings and evenings (the time points when the steps needed to be completed) to address questions or concerns. An additional consideration is data collection occurred after COVID-19 and the pandemic might have altered PA behaviour in older adults. However, in October 2021 when data collection commenced, facilities had been reopened to the public (e.g., gyms, tennis courts, walking tracks, ski resorts, etc.) in the Thompson-Okanagan region.

Participants received a $50 CDN grocery gift card for their participation which might have potentially increased recruitment of socioeconomically disadvantaged individuals. It might also have encouraged participants to remain in the study for the duration of data collection through any challenges they might have experienced (e.g., time requirements, and unfamiliar technology).

## 5 Conclusions

The present study suggests that weather might not act as a barrier to PA in older adults located in arid-regions. Generally, variability in most weather variables did not tend to impact PA and SB in older adults who typically meet recommendations of the Canadian Physical Activity guidelines (43). However, LPA engagement was associated with increased temperatures and these activities might represent daily physical activities. Interventions and programs aimed at helping older adults increase their PA may receive better uptake if offered indoors. As men are spending less time engaged in LPA and more time sedentary compared to females, indoor PA programs targeted toward males may be an important gap to fill to better reach this demographic. Future studies should further investigate the effects of weather on LPA in older adults with a larger sample size of persons who engage in low levels of PA. Future work should include more diverse populations (e.g., individuals of lower health status, different geographic regions or socioeconomic backgrounds) to enhance the generalizability and relevance of this research.

## Data Availability

The raw data supporting the conclusions of this article will be made available by the authors, without undue reservation.
